# Suitable habitat of wild Asian elephant in Western Terai of Nepal

**DOI:** 10.1002/ece3.6356

**Published:** 2020-05-25

**Authors:** Purushottam Sharma, Saroj Panthi, Subodh Kumar Yadav, Manoj Bhatta, Ajay Karki, Tom Duncan, Megharaj Poudel, Krishna Prasad Acharya

**Affiliations:** ^1^ Department of National Parks and Wildlife Conservation Kathmandu Nepal; ^2^ Ministry of Industry, Tourism, Forest, and Environment Pokhara Nepal; ^3^ Department of Survey Kathmandu Nepal; ^4^ Research Institute for Environment and Livelihoods Charles Darwin University Darwin NT Australia; ^5^ Ministry of Forests and Environment Kathmandu Nepal; ^6^ Forest Research and Training Centre Babarmahal, Kathmandu Nepal; ^7^ Ministry of Industry, Tourism, Forest, and Environment Surkhet Nepal

**Keywords:** Anthropogenic threats, habitat fragmentation, low land, variables, wildlife corridor

## Abstract

**Background:**

There is currently very little available research on the habitat suitability, the influence of infrastructure on distribution, and the extent and connectivity of habitat available to the wild Asian elephant (*Elephas maximus*). Information related to the habitat is crucial for conservation of this species.

**Methods:**

In this study, we identified suitable habitat for wild Asian elephants in the Western Terai region of Nepal using Maximum Entropy (MaxEnt) software.

**Results:**

Of 9,207 km^2^, we identified 3194.82 km^2^ as suitable habitat for wild Asian elephants in the study area. Approximately 40% of identified habitat occurs in existing protected areas. Most of these habitat patches are smaller than previous estimations of the species home range, and this may reduce the probability of the species continued survival in the study area. Proximity to roads was identified as the most important factor defining habitat suitability, with elephants preferring habitats far from roads.

**Conclusions:**

We conclude that further habitat fragmentation in the study area can be reduced by avoiding the construction of new roads and connectivity between areas of existing suitable habitat can be increased through the identification and management of wildlife corridors between habitat patches.

## Graphical abstract

Of 9,207 km^2^, we identified 3,194.82 km^2^ as suitable habitat for elephants in the study area. Approximately 40% of identified habitat occurs in existing protected areas. Proximity to roads was identified as the most important factor defining habitat suitability, with elephants preferring habitats far from roads.

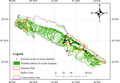

## 1 INTRODUCTION

Wild Asian elephants (*Elephas maximus*) are endangered megafauna of the tropical and subtropical regions of Asia. It is native to 13 Asian countries including Nepal and is listed as “Endangered” in the International Union for Conservation of Nature (IUCN) Red List of Threatened Species (Choudhury et al., 2008) and appendix I of the Convention on International Trade in Endangered Species of Wild Fauna and Flora (CITES, 2017). This animal is also protected by the Nepalese Government *National Parks and Wildlife Conservation Act* 1973 (GoN, 1973).

Intact rainforest fragments, riparian vegetation, and grasslands are the preferred habitats of the wild Asian elephant in India (Kumar, Mudappa, & Raman, 2010; Sukumar, 1989). In the Shivalik range of India, Kamala trees (*Mallatus philippines*) are indicator of the presence of this species during the dry season (Bi et al., 2016). In Nepal, Pradhan and Wegge (2007) described riverine forest and tall grassland as preferred habitats with *Spatholobus parviflorus* and *Saccharum spontaneum* comprising major food items (Koirala, Raubenheimer, Aryal, Pathak, & Ji, 2016).

Population of wild Asian elephant within Nepal has been estimated to be between 109 and 142 individuals (DNPWC, 2012; Pradhan, Williams, & Dhakal, 2011) with distribution concentrated in protected areas of the Terai (low land) region, in the central and eastern parts of the country, with relatively low numbers in the west (Koirala et.al., 2015).

Habitat loss, conflict with human, electrocution, and poaching are threats to elephants (Cordingley, 2008; Hoare, 1999; Kalam, Kumar Baishya, & Smith, 2018; Sampson et al., 2018; Sukumar, Ramakrishnan, & Santosh, 1998). The main threats to the survival of the wild Asian elephant are changes in the habitat and reduction in its suitable habitat, and these are caused by increased human activities (Zhang & Wang, 2003). Human expansion transforms natural habitats of wildlife into human settlements and agricultural lands (Cordingley, 2008; Hoare, 1999). Forests outside the protected areas have suffered extensive exploitation, due to the demands of human populations living along the fringe of the forest (Pradhan et al., 2011). This exploitation resulted habitat fragmentation and reduction and human–elephant conflict are frequent as elephants commonly raid crops, destroy property, and cause human injuries and fatalities (Acharya, Paudel, Neupane, & Kohl, 2016; DNPWC, 2015; Koirala, Ji, Aryal, Rothman, & Raubenheimer, 2015; Pant, Dhakal, Pradhan, Leverington, & Hockings, 2016).

This study explored on how these threats are likely to impact current populations of elephants and the extent and connectivity of suitable habitat both inside and outside protected areas. Research into these factors is therefore crucial to ensuring the species continued survival within the country. The study identified the important habitat parameters and environmental variables within topographic, vegetation related, and anthropogenic category that determine suitable wild Asian elephant habitat in the Western Terai region of Nepal.

## 2 MATERIALS AND METHODS

### 2.1 Study area

**Figure 1:**
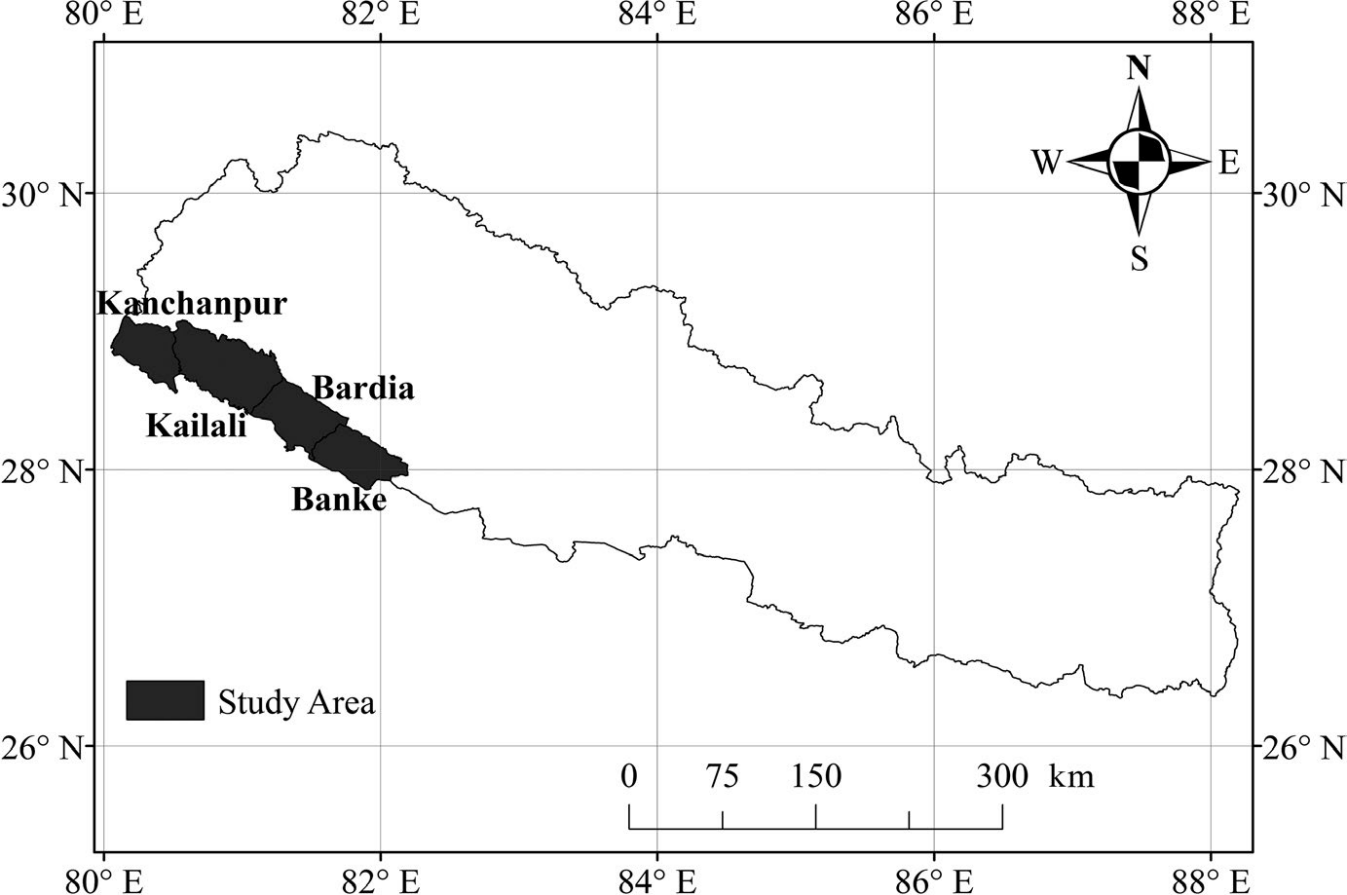
Study area in Nepal

The study was conducted in the Banke, Bardia, Kailali, and Kanchapur districts of western Nepal, with a total area of 9,207 km^2^ (Figure 1). Protected areas within the study site are Banke National Park and its Buffer Zone, Bardia National Park and its Buffer Zone, Shuklaphanta National Park and its Buffer Zone and Krishnasar Conservation Area (DNPWC, 2017). National parks belong to II and conservation area and buffer zone belong to VI according to Protected Area Categories System of International Union for Conservation of Nature (iucn.org). Entry without permission of park authority is prohibited in national parks, but reasonable entry is accepted for local people for their daily activities in buffer zones and conservation area. The lowland Terai of Nepal is an area of high biodiversity and significant conservation value. Dominant tree species in the region are sal (*Shorea robusta*)*,* asna (*Terminalia tomentosa*)*,* botdhamero (*Lagestroemia parviflora*), and sindure (*Mallatus Philippines*) (DFRS, 2015), and major fauna species include wild Asian elephant (*E. maximas*)*,* spotted deer (*Axis axis*)*,* gaur (*Bos gaurus*)*,* swamp deer (*Cervus duvaucelii*)*,* tiger (*Panthera tigris*)*,* common leopard (*P. paradus*)*,* python (*Python molurus*)*,* rhino (*Rhinoceros unicornis*)*,* sambar deer (*Rusa unicolor*)*,* wild boar (*Sus scrofa*) four‐horned antelope (*Tetracerus quadricornis*), and giant hornbill (*Buceros bicornis*) (DNPWC, 2016; Oli et al., 2018).

### 2.2 Data collection

#### 2.2.1 Elephant occurrence points

Occurrence points of wild Asian elephant were collected between September 2017 and March 2018. We first held discussions with officials responsible for protected areas in the region to identify potential habitat of elephants and visited identified areas from these discussions to record evidence of elephant presence. Elephant presence was collected through direct observation of individuals, as well as indirect observation of tracks and droppings. We also used secondary sources of elephant occurrence records, previously recorded observations (GPS points) by park authorities, in each of the protected area site offices. We collected a total of 76 records (GPS points) of elephant presence during data collection.

#### 2.2.2 Environmental variables

##### Topographical variables

Digital elevation model (DEM) data of 30 m resolution were downloaded from the United States Geological Survey website (https://earthexplorer.usgs.gov/), and the slope was computed from the DEM using ArcGIS software (ESRI, 2017). Shapefiles of water sources were downloaded from Geofabrik website (https://www.geofabrik.de/data/shapefiles.html) and converted to distance raster file using ArcGIS (ESRI, 2017). Elevation was used as a proxy of temperature due to the unavailability of high‐resolution climatic variables.

##### Vegetation‐related variables

Herbivores are depended on vegetation‐related variables (Andersen et al., 2000). The elephant is a mega herbivore, so the inclusion of vegetation‐related variables to predict suitable habitat for this species is a prerequisite for robust habitat modeling.

For the variable “forest cover,” we used data prepared by Hansen et al. (2013) which were downloaded from the Global Forest Change (GFC) website. This study used Enhanced Vegetation Index (EVI) time series data for 2015, 2016, and 2017, from images obtained by Moderate Resolution Imaging Spectroradiometer (MODIS) (https://earthexplorer.usgs.gov/).The data were then smoothed using an adaptive Savitzky‐Golay filter in the TIMESAT program (Jönsson & Eklundh, 2004), to reduce cloud cover in Environment for Visualizing Images, a software of image analysis, and the EVI values were averaged over all the indices in order to obtain the final EVI index.

##### Anthropogenic variables

Human activities have been identified as a threat to wild Asian elephants and influence the species distribution (Choudhury et al., 2008; DNPWC, 2012). We, therefore, incorporated anthropogenic variables into our model. Anthropogenic variables were the distance to human paths (used by human and animal) and roads (used by vehicle), distance to settlements, and land use. Location of paths and roads was obtained from shapefiles available on the Geofabrik website (https://www.geofabrik.de/data/shapefiles.html). Settlement locations were obtained from the Department of Survey, Nepal. Distance raster files of paths, roads, and settlements were created using ArcGIS (ESRI, 2017). Land cover and land use (LULC) data were downloaded from the International Centre for Integrated Mountain Development website (ICIMOD; http://www.icimod.org) (Uddin et al., 2015) and incorporated into the model.

### 2.3 Prediction of distribution of the wild Asian elephant

MaxEnt is a software package used to model species distributions using geo‐referenced occurrence data and environmental variables to predict suitable habitat for a species (Phillips, Anderson, & Schapire, 2006). This software extracts a sample of background locations that it contrasts against the presence locations and estimate the density of presences across the landscape (Merow, Smith, & Silander, 2013; Phillips et al., 2006). We incorporated the variables listed in Table 1 into MaxEnt (version 3.4.1) along with our occurrence data to determine habitat suitability for wild Asian elephants within our study area**.** The MaxEnt program is widely used to map wildlife habitat and identify the influence of environmental variables on species occurrence in similar study areas (Aryal et al., 2016; Bista, Panthi, & Weiskopf, 2018; KC et al., 2019; Panthi, 2018; Panthi, Wang, Sun, & Thapa, 2019; Pokharel, Ludwig, & Storch, 2016). Multicollinearity between environmental variables described in Table 1 is acceptable (|*r*| < .70) (Dormann et al., 2013), so we used all variables in the model. We maintained at least 1 km distances between species presence points to lessen spatial autocorrelation. We selected 1,000 maximum iterations and 10 replicates during modeling (Barbet‐Massin, Jiguet, Albert, & Thuiller, 2012).

**Table 1:** Environmental variables considered in the model

Category	Source	Variable	Type	Unit
Topographic	USGS	Elevation	Continuous	m
		Slope	Continuous	Degree
	GEOFABRIK	Distance to water	Continuous	m
Vegetation‐related	MODIS	Mean EVI	Continuous	Dimensionless
		Standard deviation of EVI	Continuous	Dimensionless
	GFC	Forest cover	Continuous	Dimensionless
Anthropogenic	GEOFABRIK	Distance to settlement	Continuous	m
		Distance to road	Continuous	m
		Distance to path	Continuous	m
	International Centre for Integrated Mountain Development	Land use land cover	Categorical	Dimensionless

Abbreviation: EVI, Enhanced Vegetation Index.

Accuracies of the model were accessed by two methods: threshold independent and threshold dependent. In the threshold independent method, the value of accuracy was directly obtained from the model, but in the threshold dependent method, we provided the threshold to maximize the sum of specificity and sensitivity. We used the area under the receiver–operator curve (AUC), which is automatically calculated during the modeling without using any threshold. An AUC < 0.7 denotes poor model performance, 0.7–0.9 denotes moderately useful model performance, and >0.9 denotes excellent model performance (Pearce & Ferrier, 2000). We chose true skill statistics (TSS) as the threshold dependent method. The TSS = Sensitivity + Specificity − 1 and ranges from −1 to 1, where values less than 0 indicate a performance no better than random and 1 indicates a perfect fit (Allouche, Tsoar, & Kadmon, 2006). We calculated TSS for all 10 model outputs in R software (R Core Team, 2018), and the final TSS was averaged from all ten replications (Bista et al., 2018; Jiang et al., 2014; Panthi, 2018). For species distribution models, presence‐only data threshold to maximize the TSS is recommended (Liu, White, & Newell, 2013); so, we used this threshold to convert the continuous habitat suitability map to a suitable/unsuitable binary map.

## 3 RESULTS

### 3.1 The suitable habitat of the wild Asian elephant

**Figure 2:**
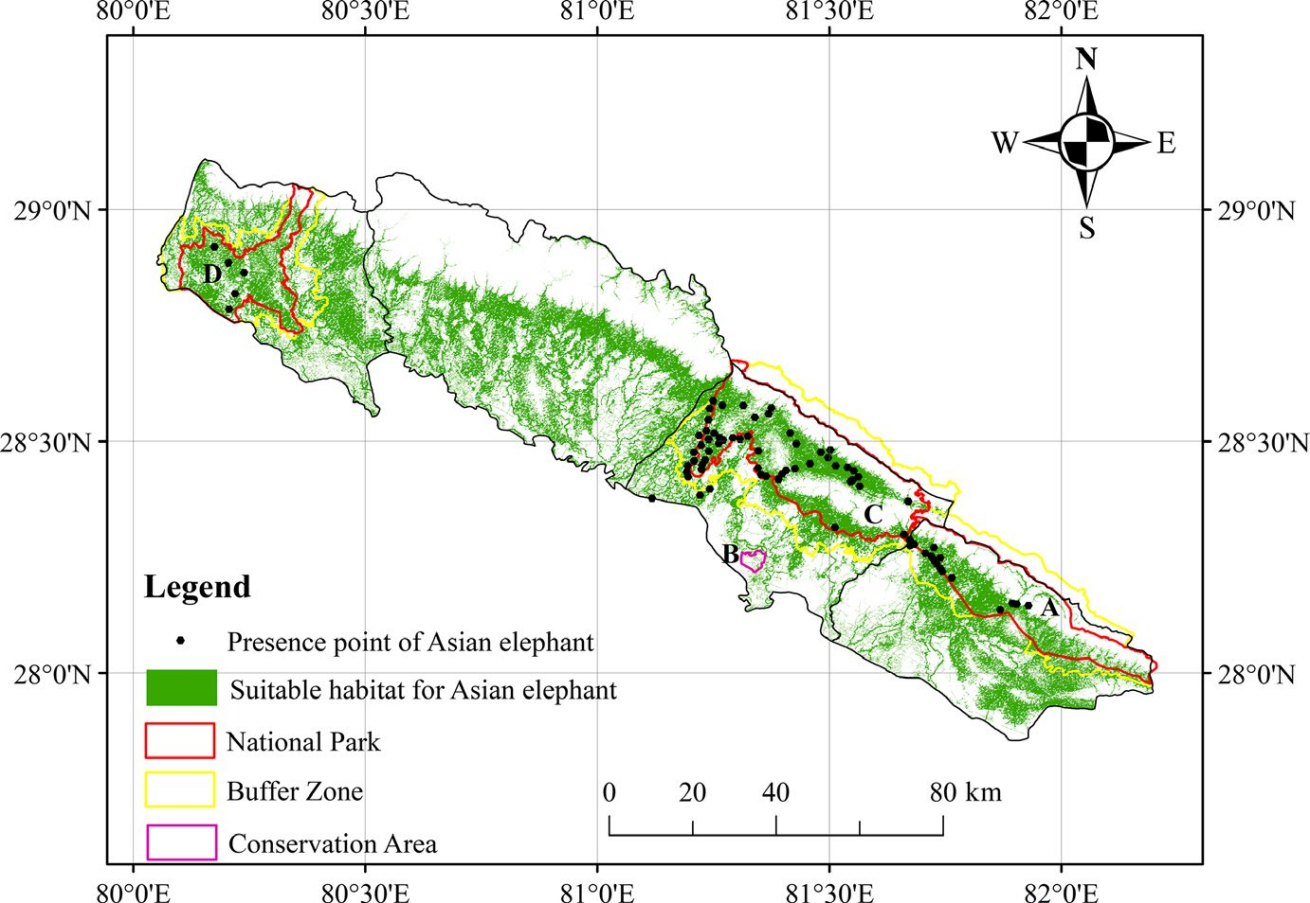
Predicted suitable habitat of wild Asian elephant based on MaxEnt modeling. (a) Banke National Park and its Buffer Zone (b) Krishnasar Conservation Area (c) Bardia National Park and its Buffer Zone (d) Shuklaphanta National Park and its Buffer Zone

We identified a total of 3,194.82 km^2^ as suitable habitat for wild Asian elephant in the study area (Figure 2). About 39.11% (1,249.58 km^2^) of this habitat occurs in existing protected areas (Table 2). Bardia National Park and its Buffer Zone contain the largest proportion of suitable habitat (46.84%), with Krishnasar Conservation Area containing the smallest portion (0.16%). The largest area of suitable habitat outside protected areas was found in Kailali district (942.55 km^2^), following Banke, Bardia, and Kanchanpur districts containing 719.46 km^2^, 798.67 km^2^, and 734.14 km^2,^ respectively. Elephant habitat in the study area was highly fragmented, occurring as small, discrete patches. Connectivity between habitat patches was low in the southern and northern parts of the study area, but higher in the center (Figure 2).

**Table 2:** Suitable habitat for wild Asian Elephant in protected areas

Protected area	Total area km^2^	Elephant's habitat km^2^
Shuklaphanta National Park and its Buffer Zone	548.5	352.09
Bardia National Park and its Buffer Zone	1,475	585.28
Banke National Park and its Buffer Zone	893	310.22
Krishnasar Conservation Area	16.95	1.99
Total	2,933.45	1,249.58

### 3.2 Important environmental variables

**Figure 3:**
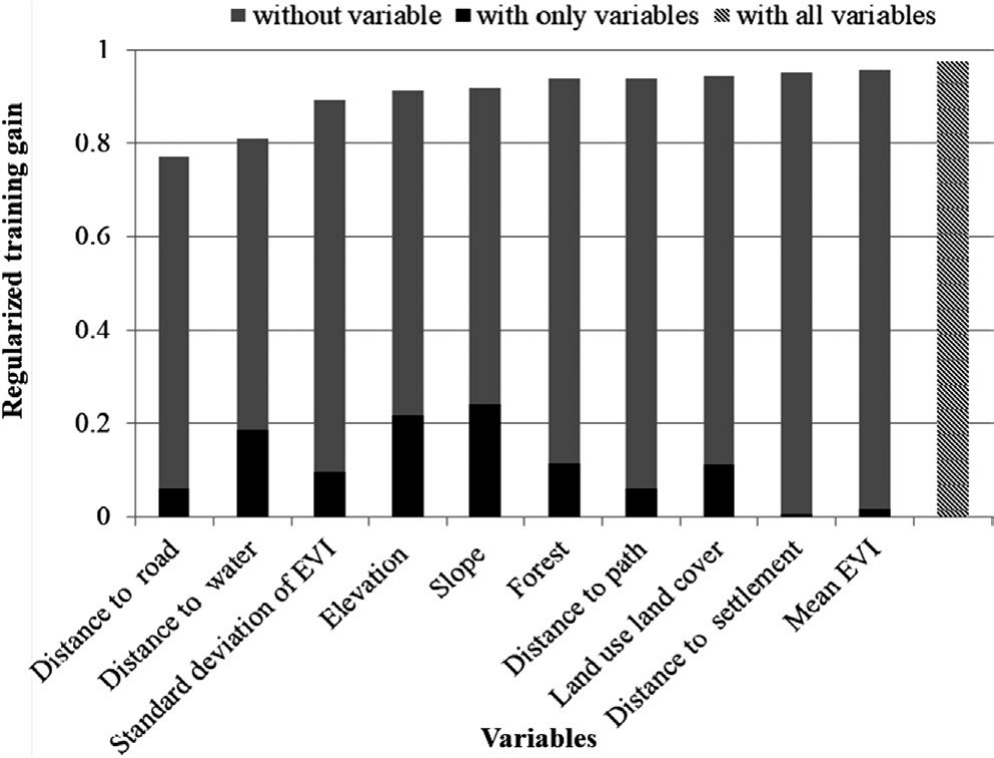
Importance of variables to train the model. The regularized training gain explains how much better the model distribution fits the presence data relative to a uniform distribution. “With all variables” indicates the results of the model when all variables are run; “with only variable” denotes the results of the model when an only that variable is run; and “without variable” denotes the effect of removing that single variable from the model (Phillips, 2017). See Table 1 for full variable names and descriptions

Of 10 variables used in the model, the distance to road, distance to water, elevation, and slope were found to be the most important variables determining habitat suitability. Distance to settlement, and mean EVI and LULC were identified as the least important variables (Figure 3). In Figure 3, the regularized training gain of the model without distance to road was less than that of the model using without other single variables, so the distance to road is a more useful variable to the model. Similarly, the regularized training gain of the models without distance to water, elevation, and slope is less, indicating that these variables are useful predictors of habitat suitability for the species.

**Figure 4:**
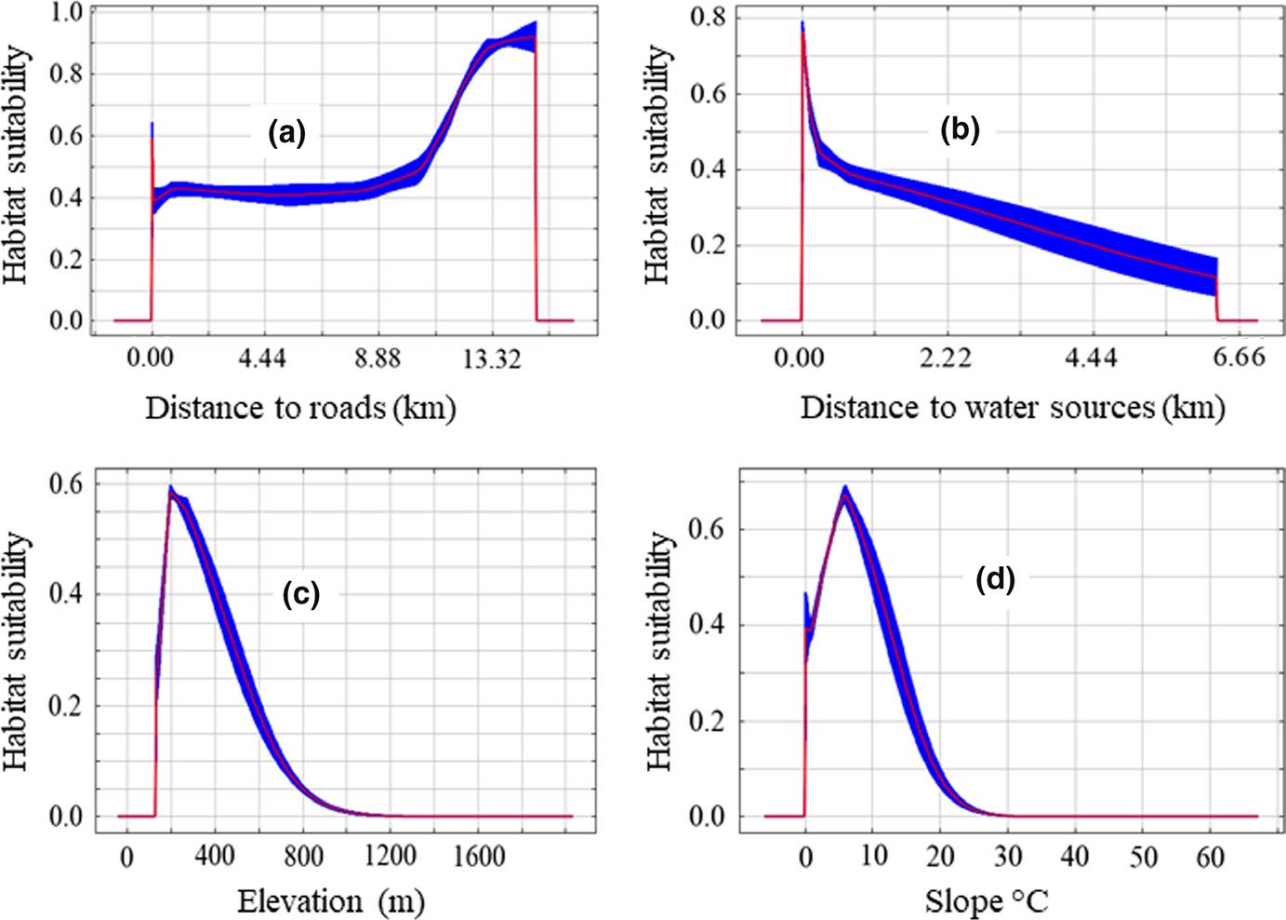
Response of habitat suitability of wild Asian elephant to variables (a) Response of habitat suitability of wild Asian elephant to distance to roads; (b) response of habitat suitability of wild Asian elephant to distance to water sources; (c) response of habitat suitability of wild Asian elephant to elevation; (d) response of habitat suitability of wild Asian elephant to slope

The model, therefore, indicates that elephants prefer habitat far from roads, near to water sources, with low elevation and gentle slope (Figure 4).

### 3.3 Model accuracy

Accuracies of the model are relatively good. We obtained 0.813 ± 0.072 AUC and 0.528 ± 0.031 TSS (Table 3). We obtained 0.214 threshold to maximize the sum of sensitivity and specificity. We used this threshold to calculate the TSS and to covert the continuous habitat suability map to binary suitable/unsuitable map.

**Table 3:** Thresholds and accuracies of different replications

S.N.	Replications												
		0	1	2	3	4	5	6	7	8	9	Average	Std
1	Threshold	0.18	0.22	0.36	0.24	0.11	0.25	0.11	0.22	0.25	0.2	0.214	0.073
3	TSS	0.545	0.596	0.556	0.58	0.418	0.543	0.402	0.595	0.578	0.47	0.528	0.072
4	AUC	0.814	0.835	0.831	0.826	0.757	0.821	0.764	0.847	0.835	0.795	0.813	0.031

## 4 DISCUSSION

Our study has identified the suitable habitat of wild Asian elephant in Banke, Bardia, Kailali, and Kanchanpur district of Nepal. Previous studies already recorded the presence of this elephant in these districts (Lamichhane et al., 2017; Neupane, Kwon, Risch, Williams, & Johnson, 2019; Pradhan & Wegge, 2007). Our result reveals most habitat (1,249.58 km^2^ or 39.11% of the total study area), located inside the protected areas where natural vegetation cover exists. Our results agree with a previous study in India which identifies the importance of natural vegetation cover to provide suitable habitat for Asian elephant (Kumar et al., 2010).

The home range size of an elephant was estimate 105–320 km^2^ in India (Sukumar, 1989). Three national parks within our study area (Banke National Park and its Buffer Zone, Bardia National Park and its Buffer Zone, and Shuklaphanta National Park and its Buffer Zone) contain a total area of suitable habitat larger than the Sukumar (1989) home range estimate (Table 2). Habitat of Asian elephant is being fragmented in China (Zhang et al., 2015). Similarly, we also find this fragmentation in our study area. Although African elephants spend much of their time in less fragmented landscapes (Gara et al., 2016), Asian elephants have been shown to continue to occur in areas with fragmented habitat (Kumar et al., 2010). Therefore, the habitats identified in these three national parks may function as significant refuges for elephants despite the fact that they occur as fragmented patches. Kailali district contains more area of suitable habitat (942.55 km^2^) outside protected areas although this district includes no protected area. Fragmented forests are more serious to human–wildlife conflict (Acharya, Paudel, Jnawali, Neupane, & Köhl, 2017). The connectivity of habitat patches in the central parts of the study area means that they have the potential to be managed as corridors to increase the likelihood of elephants moving between habitat patches and mitigate conflict between elephants and humans.

According to our model, distance to roads was found to be the major component of habitat suitability of the wild Asian elephant. Within its home range, the Asian elephant faces threats caused by increased proximity to humans, including poaching and conflict arising from human–elephant interactions such as human casualties, crop raiding and damage to property (Acharya et al., 2016, 2017; Chen et al., 2016; Choudhury et al., 2008; Gubbi, Swaminath, Poornesha, Bhat, & Raghunath, 2015; Jadhav & Barua, 2012; Lamichhane et al., 2017; Pant et al., 2016; Sukumar et al., 1998). Cultivation of traditional crops, bananas, and home alcohol production increases the chance of elephant attacks (Neupane et al., 2017). While our results indicate that elephants avoid roads, our study agrees with previous studies which described elephant presence close to other areas of human activity (Blake et al., 2008; Granados, Weladji, & Loomis, 2012; Neupane et al., 2019) such as paths and settlements. This proximity increases the likelihood of human–elephant conflict. Similar to previous studies, we found that elephant prefers habitat with low elevation, gentle slope, and proximity to water resources (Bohrer, Beck, Ngene, Skidmore, & Douglas‐hamilton, 2014; Lin et al., 2008).

## 5 CONCLUSIONS

This study identified more than 3,000 km^2^ of area as the suitable elephant habitat in the Western Terai region of Nepal. Around 40% of suitable habitat is covered by existing protected areas. Although there is large suitable habitat, the majority of suitable habitat occurs in small, discrete patches insufficient to accommodate the large resource requirements of the species. To increase connectivity between these patches, we recommend protecting existing habitat to provide corridors between Bardia National Park and Shuklaphanta National Park. The future road projects should consider the movement of wild Asian elephant and design accordingly.

## CONFLICT OF INTEREST

None declared.

## AUTHOR CONTRIBUTION

**Purushottam Sharma:** Conceptualization (equal); Data curation (lead); Project administration (lead). **Saroj Panthi:** Conceptualization (equal); Data curation (supporting); Formal analysis (lead); Investigation (lead); Methodology (lead); Software (lead); Validation (lead); Visualization (lead); Writing‐original draft (lead); Writing‐review & editing (equal). **Subodh Kumar Yadav:** Writing‐review & editing (equal). **Manoj Bhatta:** Writing‐review & editing (equal). **Ajay Karki:** Writing‐review & editing (equal). **Tom Duncan:** Writing‐review & editing (equal). **Megharaj Poudel:** Writing‐review & editing (equal). **Krishna Prasad Acharya:** Writing‐review & editing (equal).

### Open Data Badge

This article has earned an Open Data Badge for making publicly available the digitally‐shareable data necessary to reproduce the reported results. The data is available at: https://doi.org/10.5061/dryad.dncjsxkwh; https://datadryad.org/stash/share/5rTRXNhoIN1etdyMQbCIpvuxIcK7-WhpwPHZdBcuBL0.

## Data Availability

Data are available on Dryad. URL: https://datadryad.org/stash/share/5rTRXNhoIN1etdyMQbCIpvuxIcK7-WhpwPHZdBcuBL0. https://doi.org/10.5061/dryad.dncjsxkwh.

## References

[ece36356-bib-0001] CITES (2017). Appendices I, II and III, Convention on international trade in endangered species of wild fauna and flora. 10.1159/000459796 712806

[ece36356-bib-0002] Acharya, K. P. , Paudel, P. K. , Jnawali, S. R. , Neupane, P. R. , & Köhl, M. (2017). Can forest fragmentation and configuration work as indicators of human – wildlife conflict? Evidences from human death and injury by wildlife attacks in Nepal. Ecological Indicators, 80, 74–83. 10.1016/j.ecolind.2017.04.037

[ece36356-bib-0003] Acharya, K. P. , Paudel, P. K. , Neupane, P. R. , & Kohl, M. (2016). Human‐wildlife conflicts in Nepal: Patterns of human fatalities and injuries caused by large mammals. PLoS One, 11, e0161717 10.1371/journal.pone.0161717 27612174 PMC5017643

[ece36356-bib-0004] Allouche, O. , Tsoar, A. , & Kadmon, R. (2006). Assessing the accuracy of species distribution models : Prevalence, kappa and the true skill statistic (TSS). Journal of Applied Ecology, 43, 1223–1232. 10.1111/j.1365-2664.2006.01214.x

[ece36356-bib-0005] Andersen, M. C. , Watts, J. M. , Freilich, J. E. , Yool, S. R. , Wakefield, G. I. , McCauley, J. F. , & Fahnestock, P. B. (2000). Regression‐tree modelling of desert tortoise habitat in the central Mojave Desert. Ecological Applications, 10, 890–900. 10.1890/1051-0761(2000)010[0890:RTMODT]2.0.CO;2

[ece36356-bib-0006] Aryal, A. , Shrestha, U. B. , Ji, W. , Ale, S. B. , Shrestha, S. , Ingty, T. , … Raubenheimer, D. (2016). Predicting the distributions of predator (snow leopard) and prey (blue sheep) under climate change in the Himalaya. Ecology and Evolution, 6, 4065–4075. 10.1002/ece3.2196 27516864 PMC4875782

[ece36356-bib-0007] Barbet‐Massin, M. , Jiguet, F. , Albert, C. H. , & Thuiller, W. (2012). Selecting pseudo‐absences for species distribution models: How, where and how many? Methods in Ecology and Evolution, 3, 327–338. 10.1111/j.2041-210X.2011.00172.x

[ece36356-bib-0008] Bi, Y. , Roy, A. , Bhavsar, D. , Xu, J. , Wang, M. , Wang, T. , & Yang, X. (2016). Kamala tree as an indicator of the presence of Asian elephants during the dry season in the Shivalik landscape of northwestern India. Ecological Indicators, 71, 239–247. 10.1016/j.ecolind.2016.07.011

[ece36356-bib-0009] Bista, M. , Panthi, S. , & Weiskopf, S. R. (2018). Habitat overlap between Asiatic black bear Ursus thibetanus and red panda Ailurus fulgens in Himalaya. PLoS One, 13, e0203697 10.1371/journal.pone.0203697 30188937 PMC6126844

[ece36356-bib-0010] Blake, S. , Deem, S. L. , Strindberg, S. , Maisels, F. , Momont, L. , Isia, I. , … Kock, M. D. (2008). Roadless wilderness area determines forest elephant movements in the Congo Basin. PLoS One, 3, e3546 10.1371/journal.pone.0003546 18958284 PMC2570334

[ece36356-bib-0011] Bohrer, G. , Beck, P. S. A. , Ngene, S. M. , Skidmore, A. K. , & Douglas‐hamilton, I. (2014). Elephant movement closely tracks precipitation‐ driven vegetation dynamics in a Kenyan forest‐savanna landscape. Movement Ecology, 2, 2 10.1186/2051-3933-2-2 25520813 PMC4267703

[ece36356-bib-0012] Chen, Y. , Marino, J. , Chen, Y. , Tao, Q. , Sullivan, C. D. , Shi, K. , & Macdonald, D. W. (2016). Predicting hotspots of human‐elephant conflict to inform mitigation strategies in Xishuangbanna, Southwest China. PLoS One, 11, e0162035 10.1371/journal.pone.0162035 27631976 PMC5025021

[ece36356-bib-0013] Choudhury, A. , Lahiri Choudhury, D. K. , Desai, A. , Duckworth, J. W. , Easa, P. S. , Johnsingh, A. J. T. , … Wikramanayake, E. (2008). Elephas maximus [WWW Document]. IUCN Red List Threat. Species. 10.2305/IUCN.UK.2008.RLTS.T7140A12828813.en

[ece36356-bib-0014] Cordingley, M. (2008). Participatory development in Nepal: Challenges and opportunities for conservation in managing human elephant conflict. Gajah, 29, 41–44.

[ece36356-bib-0015] R Core Team (2018). R: A language and environment for statistical computing. Vienna, Austria: R Foundation for Statistical Computing.

[ece36356-bib-0016] DFRS (2015). State of Nepal’s Forests. Katthmandu, Nepal: Forest Resource Assessment (FRA), Department of Forest Research and Survey (DFRS).

[ece36356-bib-0017] DNPWC (2012). Annual Report (July 2011–July 2012). Nepal: Department of National Parks and Wildlife Conservation.

[ece36356-bib-0018] DNPWC (2015). Annual Report (July, 2014–June 2015). Nepal: Department of National Parks and Wildlife Conservation.

[ece36356-bib-0019] DNPWC (2016). Annual Report (July 2015–June 2016). Nepal: Department of National Parks and Wildlife Conservation.

[ece36356-bib-0020] DNPWC (2017). Protected areas of Nepal. Kathmandu, Nepal: Department of national parks and wildlife conservation.

[ece36356-bib-0021] Dormann, C. F. , Elith, J. , Bacher, S. , Buchmann, C. , Carl, G. , Carré, G. , … Lautenbach, S. (2013). Collinearity: A review of methods to deal with it and a simulation study evaluating their performance. Ecography, 36, 27–46. 10.1111/j.1600-0587.2012.07348.x

[ece36356-bib-0022] ESRI (2017). ArcGIS Desktop: Release 10.5. Redlands, CA: Environmental Systems Research.

[ece36356-bib-0023] Gara, T. W. , Wang, T. , Skidmore, A. K. , Zengeya, F. M. , Ngene, S. M. , Murwira, A. , & Ndaimani, H. (2016). Understanding the effect of landscape fragmentation and vegetation productivity on elephant habitat utilization in Amboseli ecosystem, Kenya. African Journal of Ecology, 55(3), 259–269. 10.1111/aje.12346

[ece36356-bib-0024] GoN (1973). National parks and wildlife conservation act. Nepal: Government of Nepal, Nepal Law Commission.

[ece36356-bib-0025] Granados, A. , Weladji, R. B. , & Loomis, M. R. (2012). Movement and occurrence of two elephant herds in a human‐dominated landscape, the Bénoué Wildlife Conservation Area, Cameroon. Tropical Conservation Science, 5, 150–162. 10.1177/194008291200500205

[ece36356-bib-0026] Gubbi, S. , Swaminath, M. H. , Poornesha, H. C. , Bhat, R. , & Raghunath, R. (2015). An elephantine challenge : Human‐elephant conflict distribution in the largest Asian elephant population, southern India. Biodiversity and Conservation, 23, 633–647. 10.1007/s10531-014-0621-x

[ece36356-bib-0027] Hansen, M. C. , Potapov, P. V. , Moore, R. , Hancher, M. , Turubanova, S. A. , & Tyukavina, A. (2013). High‐resolution global maps of 21st‐century forest cover change. Science, 342(6160), 850–853. 10.1126/science.1244693 24233722

[ece36356-bib-0028] Hoare, R. E. (1999). Determinants of human‐elephant conflict in a land‐use mosaic. Journal of Applied Ecology, 36, 689–700. 10.1046/j.1365-2664.1999.00437.x

[ece36356-bib-0029] Jadhav, S. , & Barua, M. (2012). The elephant vanishes: Impact of human – elephant conflict on people ’ s wellbeing. Health Place, 18, 1356–1365. 10.1016/j.healthplace.2012.06.019 22819603

[ece36356-bib-0030] Jiang, Y. , Wang, T. , De Bie, C. A. J. M. , Skidmore, A. K. , Liu, X. , Song, S. , … Shao, X. (2014). Satellite‐derived vegetation indices contribute significantly to the prediction of epiphyllous liverworts. Ecological Indicators, 38, 72–80. 10.1016/j.ecolind.2013.10.024

[ece36356-bib-0031] Jönsson, P. , & Eklundh, L. (2004). TIMESAT ‐ A program for analyzing time‐series of satellite sensor data. Computers & Geosciences, 30, 833–845. 10.1016/j.cageo.2004.05.006

[ece36356-bib-0032] Kalam, T. , Kumar Baishya, H. , & Smith, D. (2018). Lethal fence electrocution: A major threat to Asian elephants in Assam, India. Tropical Conservation Science, 11, 1–8. 10.1177/1940082918817283

[ece36356-bib-0033] Kc, K. B. , Koju, N. P. , Bhusal, K. P. , Low, M. , Ghimire, S. K. , Ranabhat, R. , & Panthi, S. (2019). Factors influencing the presence of the endangered Egyptian vulture Neophron percnopterus in Rukum, Nepal. Global Ecology and Conservation, 20, e00727 10.1016/j.gecco.2019.e00727

[ece36356-bib-0034] Koirala, R. K. , Ji, W. , Aryal, A. , Rothman, J. , & Raubenheimer, D. (2015). Dispersal and ranging patterns of the Asian Elephant (*Elephas maximus*) in relation to their interactions with humans in Nepal. Ethology Ecology & Evolution, 28, 1–12. 10.1080/03949370.2015.1066872

[ece36356-bib-0035] Koirala, R. K. , Raubenheimer, D. , Aryal, A. , Pathak, M. L. , & Ji, W. (2016). Feeding preferences of the Asian elephant (Elephas maximus) in Nepal. BMC Ecology, 16, 54 10.1186/s12898-016-0105-9 27855704 PMC5114758

[ece36356-bib-0036] Kumar, M. A. , Mudappa, D. , & Raman, T. R. S. (2010). Asian elephant Elephas maximus habitatuse and ranging in fragmented rainforest and plantations in the Annamalai Hills, India. Tropical Conservation Science, 3, 143–158.

[ece36356-bib-0037] Lamichhane, B. R. , Subedi, N. , Pokheral, C. P. , Dhakal, M. , Acharya, K. P. , Pradhan, N. M. B. , … Yackulic, C. B. (2017). Using interviews and biological sign surveys to infer seasonal use of forested and agricultural portions of a human‐dominated landscape by Asian elephants in Nepal. Ethology Ecology & Evolution, 30(4), 331–347. 10.1080/03949370.2017.1405847

[ece36356-bib-0038] Lin, L. , Feng, L. , Pan, W. , Guo, X. , Zhao, J. , Luo, A. , & Zhang, L. I. (2008). Habitat selection and the change in distribution of Asian elephants in Mengyang Protected Area, Yunnan, China. Acta Oecologica, 53, 365–374. 10.1007/BF03195197

[ece36356-bib-0039] Liu, C. , White, M. , & Newell, G. (2013). Selecting thresholds for the prediction of species occurrence with presence‐only data. Journal of Biogeography, 40, 778–789. 10.1111/jbi.12058

[ece36356-bib-0040] Merow, C. , Smith, M. J. , & Silander, J. A. (2013). A practical guide to MaxEnt for modeling species’ distributions: What it does, and why inputs and settings matter. Ecography, 36, 1058–1069. 10.1111/j.1600-0587.2013.07872.x

[ece36356-bib-0041] Neupane, D. , Johnson, R. L. , & Risch, T. S. (2017). How do land‐use practices affect human—elephant conflict in nepal? Wildlife Biology, 2017(1), wlb.00313 10.2981/wlb.00313

[ece36356-bib-0042] Neupane, D. , Kwon, Y. , Risch, T. S. , Williams, A. C. , & Johnson, R. L. (2019). Habitat use by Asian elephants: Context matters. Global Ecology and Conservation, 17, e00570 10.1016/j.gecco.2019.e00570

[ece36356-bib-0043] Oli, C. B. , Panthi, S. , Subedi, N. , Ale, G. , Pant, G. , Khanal, G. , & Bhattarai, S. (2018). Dry season diet composition of four‐ horned antelope Tetracerus quadricornis in tropical dry deciduous forests, Nepal. Peerj, 6, e5102 10.7717/peerj.5102 29967747 PMC6022733

[ece36356-bib-0044] Pant, G. , Dhakal, M. , Pradhan, N. M. B. , Leverington, F. , & Hockings, M. (2016). Nature and extent of human–elephant Elephas maximus conflict in central Nepal. Oryx, 50, 724–731. 10.1017/S0030605315000381

[ece36356-bib-0045] Panthi, S. (2018). Predicting current and future habitat suitability for red pandas in Nepal. MSc thesis. University of Twente, Faculty of Geoinformation and Earth Observation, Enschede, Netherlands.

[ece36356-bib-0046] Panthi, S. , Wang, T. , Sun, Y. , & Thapa, A. (2019). An assessment of human impacts on endangered red pandas (*Ailurus fulgens*) living in the Himalaya. Ecology and Evolution, 9, 13413–13425. 31871654 10.1002/ece3.5797 PMC6912920

[ece36356-bib-0047] Pearce, J. , & Ferrier, S. (2000). Evaluating the predictive performance of habitat models developed using logistic regression. Ecological Modelling, 133(3), 225–245. 10.1016/S0304-3800(00)00322-7

[ece36356-bib-0048] Phillips, S. J. (2017). A brief tutorial on Maxent. 10.4016/33172.01

[ece36356-bib-0049] Phillips, S. J. , Anderson, R. P. , & Schapire, R. E. (2006). Maximum entropy modelling of species geographic distributions. Ecological Modelling, 190, 231–259. 10.1016/j.ecolmodel.2005.03.026

[ece36356-bib-0050] Pokharel, K. P. , Ludwig, T. , & Storch, I. (2016). Predicting potential distribution of poorly known species with small database: The case of four‐horned antelope Tetracerus quadricornis on the Indian subcontinent. Ecology and Evolution, 6, 2297–2307. 10.1002/ece3.2037 27069584 PMC4782261

[ece36356-bib-0051] Pradhan, N. M. B. , & Wegge, P. (2007). Dry season habitat selection by a recolonizing population of Asian elephants Elephas maximus in lowland Nepal. Acta Theriologica, 52(2), 205–214. 10.1007/BF03194216

[ece36356-bib-0052] Pradhan, N. M. B. , Williams, A. C. , & Dhakal, M. (2011). Current status of Asian Elephants in Nepal. Gajah, 35, 87–92.

[ece36356-bib-0053] Sampson, C. , McEvoy, J. , Oo, Z. M. , Chit, A. M. , Chan, A. N. , Tonkyn, D. , … Leimgruber, P. (2018). New elephant crisis in Asia ‐ Early warning signs from Myanmar. PLoS One, 13, e0194113 10.1371/journal.pone.0194113 29534096 PMC5849331

[ece36356-bib-0054] Sukumar, R. (1989). Ecology of the asian elephant in southern india. i. movement and habitat utilization patterns. Journal of Tropical Ecology, 5, 1–18. 10.1017/S0266467400003175

[ece36356-bib-0055] Sukumar, R. , Ramakrishnan, U. , & Santosh, J. A. (1998). Impact of poaching on an Asia elephant population in southern India. Animal Conservation, 1, 281–291.

[ece36356-bib-0056] Uddin, K. , Shrestha, H. L. , Murthy, M. S. R. , Bajracharya, B. , Shrestha, B. , Gilani, H. , … Dangol, B. (2015). Development of 2010 national land cover database for the Nepal. Journal of Environmental Management, 148, 82–90. 10.1016/j.jenvman.2014.07.047 25181944

[ece36356-bib-0057] Zhang, L. , Dong, L. , Lin, L. , Feng, L. , Yan, F. , Wang, L. , … Luo, A. (2015). Asian elephants in China: Estimating population size and evaluating habitat suitability. PLoS One, 10, e0124834 10.1371/journal.pone.0124834 25992617 PMC4438002

[ece36356-bib-0058] Zhang, L. , & Wang, N. (2003). An initial study on habitat conservation of Asian elephant (*Elephas maximus*), with a focus on human elephant conflict in Simao, China. Biological Conservation, 112, 453–459. 10.1016/S0006-3207(02)00335-X

